# Study protocol for applying flourishing to healthy aging: a cross-cultural exploration of older adults’ lived experiences and experts’ perspectives

**DOI:** 10.3389/fpsyg.2026.1849648

**Published:** 2026-09-23

**Authors:** Sneha Ouseph, Sara Doménech, Ruth Angiel Padiri, Ramon Miralles-Basseda, Ana Barajas

**Affiliations:** 1 Fundació Salut i Envelliment (Foundation on Health and Ageing), Universitat Autònoma de Barcelona, Barcelona, Spain; 2 Universitat Autonoma de Barcelona Departament de Psicologia Clinica i de la Salut, Bellaterra, Spain; 3 Department of Applied Psychology, Central University of Tamil Nadu, Tiruvarur, India; 4 Department of Medicine, Universitat Autonoma de Barcelona, Bellaterra (Cerdanyola del Valles), Spain; 5 Serra Húnter Programme, Government of Catalonia, Barcelona, Spain

**Keywords:** flourishing, healthy aging, older adults, protective factors, psychosocial well being, qualitative cross-cultural research, quality of life, stakeholders

## Abstract

Healthy aging is often misinterpreted as merely delaying biological aging and extending longevity, overlooking the broader dimensions of wellbeing and quality of life in later years. Dominant biomedical frameworks frequently underestimate the human essence in aging, neglecting the holistic and multidimensional aspects of life that contribute to thriving in late adulthood. This study applies the concept of flourishing to examine healthy aging by integrating Martin Seligman’s PERMA model of well-being and Ryff’s six-dimensional model of psychological wellbeing. The purpose of this research is to explore the concept and process of aging by comparing the lived experiences of older adults with the perspectives of stakeholders involved in older adult care across two cultural contexts: India and Spain. Specifically, the study aims to identify factors and psychosocial protective elements that support flourishing, quality of life, and cognitive well-being in late adulthood, and to compare these factors across two cultures. A total of thirty older adults and thirty stakeholders will be recruited across the two countries using purposive sampling. Semi-structured in depth interviews will be conducted, guided by phenomenological inquiry for older adults and interpretative inquiry for stakeholders, using separate interview guides for each group. Data will be analyzed using reflexive thematic analysis. Findings are expected to provide insights into person-centered, culturally sensitive, non-pharmacological interventions and policies that support holistic well-being in later life. This study contributes to reframing aging as a process of “growing old well,” emphasizing well-being and quality of life across the lifespan.

## 1 Introduction

The United Nations has declared 2021–2030 as the Decade of Healthy Aging, emphasizing the need for actions that enhance the lives of older adults in light of the rapidly growing older population worldwide. However, dominant fields such as medicine have long bio medicalized the process of aging, leading to the social construction of old age as a period of loss and decline (Estes and Binney, 1989). In contrast, the World Health Organization (World Health Organization: WHO, 2020) emphasizes that healthy aging involves developing and maintaining the functional ability that enables well-being in older age. In addition, the World Health Organization: WHO (2012) stated that the goal is to “add life to years, not just years to life,” underscoring the importance of quality of life in discussions of healthy aging. The previous studies, such as Ryff (1989), indicate the neglect of human essence in aging studies and the insufficient attention given to positive perspectives on the aging process.

In this context, the field of psychology becomes particularly significant, as it offers a new scope for redefining aging in an optimistic, non-pharmacological, and person-centered way. The lifespan developmental approach (Baltes, 1987) states that human development is a continuous process throughout the lifespan, from birth to death, influenced by biological, psychological, and social factors, and involving both gains and losses at each stage. In contrast, theories of well-being in positive psychology developed by Seligman (2011) and Ryff (1989) describe the importance of flourishing—an optimal state of living well at every stage of life—beyond the mere absence of illness. Together, these approaches provide scope to redefine aging as growing old with the potential to live well in later years of life, aligning with the WHO’s assertion that everyone can experience healthy aging and that being free of disease or infirmity is not a requirement for healthy aging.

Flourishing in late adulthood refers to making the most out of life despite the challenges that accompany aging (Sorrell, 2017). Seligman (2011) proposes that flourishing is possible at any stage of life, including late adulthood, through five core pillars—PERMA—encompassing positive emotions, engagement, meaningful relationships, meaning in life, and a sense of accomplishment. Empirical work applying the PERMA framework in later life suggests that it provides a useful lens for understanding healthy aging. For example, Waterworth et al. (2019) demonstrated that older adults’ well-being can be meaningfully explored through PERMA, while also highlighting that risks to well-being may arise from contextual factors such as environmental and financial constraints that limit individuals’ capacity to engage in positive actions. Their findings further emphasized the importance of fostering conversations with older adults about well-being and co-developing practices that are personally meaningful to them.

In contrast, Ryff (2013) conceptualizes human flourishing in terms of psychological well-being, emphasizing autonomy, environmental mastery, personal growth, positive relations with others, purpose in life, and self-acceptance. Similarly, quality of life is defined as an individual’s subjective perception of their position in life, shaped by their cultural context, value systems, goals, expectations, standards, and concerns (WHOQOL Group, 1995). Together, these constructs provide a comprehensive framework for understanding well-being in later life: flourishing captures the multidimensional, subjective experience of living well, while quality of life reflects individuals’ evaluations of their life circumstances within broader cultural and structural contexts.

Cognitive well-being in this study is conceptualized as a subjective and functional construct emerging from participants’ accounts of daily activities, cognitive engagement, and perceived mental functioning, as described through their lived experiences (Huang et al., 2026). According to the National Institute on Aging (2024), cognitive health refers to the ability to think, learn, and remember clearly. Previous studies have demonstrated a significant association between flourishing, quality of life, and cognitive well-being in later life, suggesting a bidirectional relationship among these constructs. A review by Zahodne (2021) highlights that the psychosocial protective factors—such as perceived control, social relationships, and religious involvement—are positively associated with cognitive health in later life. Research by Lv et al. (2025) indicates that higher levels of flourishing are associated with a lower risk of mild cognitive impairment, partly through the enhancement of psychological resilience. Similarly, Stites et al. (2018) found that subjective cognitive complaints are linked to poorer quality of life and increased psychological distress, including depression, anxiety, and stress, thereby negatively affecting overall mental well-being. Together, these findings highlight the interconnected role of flourishing, quality of life, and cognitive health in promoting healthy aging.

Research demonstrates that the meaning of healthy aging is not universal but shaped by cultural contexts. Hung et al. (2010), in their comparison of Western and non-Western perspectives, argue that healthy aging is a culturally constructed concept. Their study revealed notable discrepancies between academic and lay views: while academics tend to emphasize biomedical and functional aspects, lay perspectives highlight broader life domains such as family, spirituality, and financial security. Similarly, Cole and Ray (2011) discussed humanistic gerontology, emphasizing the need to study aging as a human process through a subjective lens. This divergence underscores the need for cross-cultural research that takes into account the lived realities and values of older adults in different societies. The study by Ayoubi-Ayoubi-Mahani et al. (2023) further supports that incorporating the perspectives of older adults and geriatric experts is crucial for designing appropriate policies and interventions that address active aging needs.

Although both India and Spain are experiencing rapid population ageing, they represent distinct structural and cultural contexts for growing older, making their comparison particularly valuable for theory-building on flourishing in later life. The rationale for this comparison is grounded in three key dimensions. First, welfare and care structures differ substantially, with Spain representing a formalized welfare state characterized by institutionalized support systems for older adults, while India continues to rely predominantly on informal, family-based caregiving arrangements. Second, intergenerational dynamics vary across contexts, reflecting the transformation of traditional joint family systems in India and the prevalence of more autonomous, yet emotionally connected, family structures in Spain. Third, meaning-making frameworks surrounding ageing, life purpose, and later-life transitions are shaped by distinct cultural, spiritual, and philosophical traditions in each setting. By examining these dimensions, the study seeks to explore how differing structural and normative environments give rise to both convergent and divergent pathways toward psychological flourishing and cognitive well-being, rather than reducing the comparison to a binary cultural contrast.

Considering the insights and limitations identified in existing literature, there is a growing need to explore healthy aging through a humanistic, holistic, and positive paradigm. This study protocol contributes to the field by outlining a structured approach to examining healthy aging through the lived experiences and perspectives of both older adults and care stakeholders across cultural contexts. By publishing the protocol in advance, the study enhances methodological transparency, rigor, and replicability in qualitative cross-cultural research. Furthermore, it demonstrates how well-being theories and lifespan developmental perspectives can be systematically integrated into healthy aging research beyond predominantly biomedical or institutional approaches. In doing so, this protocol aims to support the advancement of theoretically grounded and culturally sensitive approaches to understanding what constitutes living well in later years.

### 1.1 Primary objective

The primary objective of this research is to study the concept and process of healthy aging from a holistic perspective by comparing the experiences of older adults and perspectives of stakeholders involved in older adult care across two cultures, India and Spain.

### 1.2 Specific objectives

The specific objectives of this research are;

- To identify factors that enhance flourishing, quality of life, and cognitive well-being in late adulthood in India and Spain based on the lived experiences of older adults.
- To identify factors that enhance flourishing, quality of life, and cognitive well-being in late adulthood in India and Spain from the perspectives of stakeholders involved in older adult well-being and care.
- To examine the commonalities and differences between older adults’ lived experiences and stakeholders’ perspectives in understanding the meaning of healthy aging.
- To examine the commonalities and differences in the determinants of healthy aging across the two cultural contexts of India and Spain.

## 2 Method

### 2.1 Research design

This study adopts an interpretivist qualitative cross-cultural design. A phenomenological lens is applied to explore older adults’ lived experiences of well-being, while an interpretivist lens guides the exploration of stakeholders’ meaning making. Both datasets will be analyzed using reflexive thematic analysis (RTA).

### 2.2 Participants

There are two populations in this current study. The first population includes 30 stakeholders involved in older adult care, with 15 participants from India and 15 from Spain. Purposive sampling will be employed to ensure diversity and richness of the data.

The proposed sample size of 15 participants per group was selected as a planning estimate, while also allowing flexibility to address practical challenges such as participant withdrawal or unavailability. In RTA (Braun and Clarke, 2019), themes are generated through the active engagement and interpretive work of the researcher, and meaning making is considered an ongoing process. Therefore, data are not viewed as becoming “saturated” in the traditional sense. Instead, emphasis is placed on the richness, depth, and relevance of the data in addressing the research objectives.

The stakeholders can be researchers, gerontologists, geriatricians, caregivers, and representatives from organizations supporting older adults. They will be recruited from across the respective countries in both cultural contexts.

Stakeholders in both countries will be identified through multiple sources, including reviews of healthy aging literature, healthy aging institutes, professional and casual networks, social media platforms, and academic conferences. Participants will be contacted and recruited through in-person meetings, phone calls, and email correspondence, with several follow-ups as required.

### 2.3 Inclusion and exclusion criteria for stakeholders

Stakeholders will be included in the study if they are actively involved in the care, support, or service provision for older adults in formal or informal roles and are able to provide informed consent. They should have direct and sustained interaction with older adults and be able to reflect on and articulate their perspectives regarding ageing, care, and well-being beyond routine task-based responsibilities.

However, stakeholders will be excluded if they have minimal or no direct interaction with older adults, or if they are engaged exclusively in highly specialised technical or biomedical roles that do not involve broader engagement with the psychosocial or well-being dimensions of ageing.

The second population includes a sample of 30 older adults, with 15 participants from India and 15 from Spain. According to World Health Organization, (2025), Older adults are defined as individuals aged 60 years and above. Older adults will be identified through multiple sources in both countries. In India, participants will be identified through social networks, family members, and friends, and will be primarily contacted and recruited through in-person meetings, and additionally via phone calls if necessary. In Spain, older adults will be identified through social networks, socio-cultural centers, and community centers, and will be contacted and recruited through in-person meetings and emails.

Older adult participants will be recruited from the state of Kerala in India and from the Catalunya region in Spain. These sites have been selected based on considerations of feasibility and access for in-depth qualitative data collection, including established local networks, language accessibility, and logistical practicality for conducting fieldwork. While the findings are not intended for statistical generalisation, the selected sites are expected to provide rich, contextually grounded insights relevant to understanding healthy ageing processes. Purposive sampling will be employed to ensure diversity and richness of the data. When selecting older adult participants in both countries, efforts will be made to include diversity in gender, socioeconomic backgrounds, and geographic locations (urban and rural). Consistent with RTA, such heterogeneity is not treated as a variable to be controlled but rather as part of the contextual richness of lived experience that informs interpretive analysis.

### 2.4 Inclusion and exclusion criteria for older adults

The older adults (young-old, old-old, oldest-old; Santrock, 2010) will be included in the study if they are 60 years and above, as well as able to express willingness to participate voluntarily and provide informed consent, irrespective of their place of residence (e.g., community dwelling, residential care, or institutional settings), in order to ensure diversity of the sample. However, older adults will be excluded if they present with severe cognitive, sensory, psychiatric, or physical impairments that would significantly limit their ability to participate meaningfully in interviews or complete study procedures, or if they are not native residents of the respective cultural context (i.e., individuals who have migrated from another country and do not have long-term residence in the study setting), in order to ensure that the sample reflects locally situated experiences of ageing within each cultural context.

### 2.5 Data collection

Data will be collected through semi-structured, in-depth interviews, enabling a comprehensive exploration of both stakeholders’ perspectives and older adults’ lived experiences related to healthy aging. Separate semi-structured interview guides were developed for each group within an overarching interpretivist qualitative framework (Table 1, Table 2). A phenomenological lens informed the older adults’ guide, while an interpretivist lens informed the stakeholders’ guide.

**Table 1:** Interview guide for stakeholders.

Interview questions	Expected themes
Could you please share briefly how you came to work in this field, and tell me a little about your journey in caring for or working with older adults?	Background, perspectives, attitudes and values of stakeholders in general
When people enter the later half of life, what do you think are the ups and downs they usually experience?	Perceived challenges and opportunities in later life; developmental transitions; gains and losses in aging
Have you noticed any routines, habits, or lifestyle qualities in older adults who seem to function positively and live well? Are there also special qualities you have observed in people who stay mentally sharp even in the later years of life?	Flourishing indicators including lifestyle, environmental and economic aspects. Cognitive wellbeing.
When people grow older, what do you feel tends to matter the most to them?	Priorities in later life and core values
What shifts have you noticed in the way older adults connect with family, friends, or community?	Support system, social relationships and connections
In your experience, what supports older adults in finding joy and peace despite the difficulties of aging?	Positive emotions, self acceptance and adaptation
What makes it possible for someone to continue living with independence and choice in later life?	Autonomy and independence
From your experience, what supports people in adjusting to the changes of later life while still moving ahead positively?	Environmental mastery, resilience and personal growth
As people grow older, they may experience losses and become more aware of life’s limits. What do you feel helps them accept themselves and live meaningfully despite this?	Self acceptance, meaning and purpose in life
What do you feel gives people the reason to keep going as they grow older?	Meaning and purpose in life
What do you think supports people in later life to keep learning, growing, and staying open, even when challenges arise?	Engagement, personal growth

**Table 2:** Interview guide for older adults.

Interview questions	Expected themes
Can you tell me a little about your life — where you were born, your family, and what your early life was like?	Lifestory in brief
What does a typical day look like for you now?	Daily routine, habits and engagement
What are your favorite hobbies or activities, and what makes you happy in your daily life?	Positive emotion
What has been the most difficult situation or challenge in your life? How did you manage it and move forward?	Personal growth, environmental mastery, and resilience
What is the most beautiful or meaningful memory in your life? Why does it stand out to you?	Meaning in life and life satisfaction
Who are the people you can rely on, and whom do you feel can rely on you?	Support system, relationships, connection and belongingness
What are your dreams at this stage of your life?	Purpose in life, future orientation and accomplishment
Do you have any regrets in life, and if so, how have you dealt with or reflected on them?	Self acceptence
At this stage of life, what do you feel are the most important things, and why?	Core values and priorities
Looking back, how do you feel about life overall and the person you have become?	Overall wellbeing and self evaluation

While two analytic lenses inform the framing of data collection, all interview data will be analyzed using RTA, allowing a consistent analytic approach across datasets. Integration will occur at the level of theme development, where themes derived from older adults’ accounts and stakeholders’ accounts will be compared and interpreted analytically to identify areas of convergence, divergence, and complementarity in understandings of well-being. Importantly, this comparative process does not assume the two groups produce equivalent forms of knowledge. Rather, it recognizes that older adults’ accounts reflect lived experience, while stakeholders’ accounts reflect professional interpretations, and these are brought into dialogue through an interpretive rather than equivalence-based comparison.

The interview questions are grounded in the theoretical framework of the study, integrating Seligman’s PERMA model and Ryff’s psychosocial dimensions. Stakeholders’ interviews will begin with questions about their professional background, roles, and strategies or interventions used, gradually moving to their perspectives on older adults’ lives. Questions also will explore the habits, patterns, and lifestyle qualities of individuals who live well in later years, beyond psychosocial and spiritual dimensions.

For older adults, interviews will begin with open-ended questions about their background, place of birth, occupation, and life history, followed by inquiries into their typical daily routines, hobbies, valued activities, and reflections on their past and future. These questions were informed by the PERMA model and Ryff’s six-dimensional model of psychological well-being to ensure conceptual coverage while remaining open-ended and flexible.

These domain labels of the theoretical models were not used as analytic categories but served only as conceptual guides for question development. The semi-structured format allows participants to reflect and share what they considered important, rather than being guided solely by the researcher. The average duration of stakeholders’ interviews may range from 30 to 50 min, while interviews with older adults are expected to last approximately 50 to 90 min. Interview length will vary depending on individual participants and the depth of discussion.

### 2.6 Data analysis

Data will be analyzed using thematic analysis, following the six-phase approach outlined by Braun and Clarke (2006). Braun and Clarke (2006) define thematic analysis as a method for identifying, analyzing, and reporting patterns or themes within qualitative data.

The analysis will begin with transcription of the interview audios in English, Malayalam, and Spanish using relevant software such as Turbo AI. Transcripts conducted in Malayalam and Spanish will be translated into English to ensure a consistent analytic language across datasets. Translation will be treated as part of the analytic process rather than a purely technical step, with attention to preserving contextual and culturally embedded meanings. This transcription and repeated reading of the verbatim data constitute the first phase of familiarization, allowing the researcher to immerse herself in the data.

Thematic analysis will be conducted using RTA. While coding will remain grounded in the data, interpretation will be informed by sensitizing concepts from the PERMA model and Ryff’s framework without imposing pre-defined categories. Coding will be conducted manually and with the support of digital tools such as Google Docs.

During the third phase, the initial codes will be organized into potential themes. In the fourth phase, these themes will be reviewed and refined to ensure internal coherence and clear distinctions. Similar themes may be collapsed, while broad themes may be divided into sub-themes as needed.

In the fifth phase, themes will be clearly defined and named, grounded primarily in participants’ narratives, with the theoretical framework used as an interpretive lens during analysis and discussion. Finally, in the sixth phase, the analysis will be finalized with a coherent write-up of the themes, integrating participants’ perspectives and cross-cultural comparisons between India and Spain, as well as between stakeholders and older adults.

Furthermore, qualitative rigor will be ensured through researcher reflexivity, regular discussions within the research team, transparent documentation of the analytical process, decisions, and insights, as well as a detailed positionality statement. Given the study adopts RTA (Braun and Clarke, 2016, 2019, 2020), the researcher’s subjectivity is considered a strength rather than a source of bias.

### 2.7 Study procedure (in steps)

- Review of Literature - A comprehensive review of the literature was conducted on healthy aging, the psychology of aging, lifespan developmental theories, well-being theories, qualitative research methodologies, and cross-cultural studies of aging. This review helped establish the theoretical foundation of the present study and identify key insights and gaps within existing research.
  The literature review also informed the selection of India and Spain as the cultural contexts for comparison. While healthy aging has been widely examined within Western frameworks, relatively fewer studies have explored the concept from non-Western perspectives. Furthermore, the review of literature confirmed that the current study is the first study on healthy aging by comparing India and Spain. India represents a rapidly aging population (World Health Organization: WHO, 2020) within a predominantly family-based caregiving system, whereas Spain is characterized by one of the highest life expectancies globally (Eurostat, 2023) and a more formalized welfare structure. Comparing these two contexts provides a meaningful opportunity to explore how healthy aging is conceptualized across diverse sociocultural and institutional settings.
- Development of interview guides - Separate semi-structured interview guides were developed for stakeholders and older adults. The development process involved multiple revisions and was theoretically informed by the PERMA model of well-being and Ryff’s six-dimensional model of psychological well-being. These frameworks guided the formulation of questions to ensure alignment with key domains of flourishing and holistic aging.
  To enhance content validity and clarity, the interview questions were reviewed by senior researchers within the research team for relevance, coherence, and alignment with the study objectives. In addition, the interview guide was pilot tested with a small group representative of the target population to assess clarity, cultural appropriateness, and comprehensibility. Feedback from both expert review and pilot testing was used to refine and finalize the interview questions prior to data collection.
    Each interview guide consists of ten open-ended questions. While the guides provide a structured framework, flexibility is maintained to allow the conversation to unfold naturally and to probe emerging themes where relevant.
    For stakeholders, the interview begins with an opening question inviting participants to briefly share their professional journey and how they came to work in the field of older adult care. This serves as an icebreaker and establishes rapport. The interview then transitions into the main thematic sections addressing perceptions of healthy aging, and wellbeing in later years. The overall approach is conversational and reflective, encouraging collaborative meaning-making between interviewer and participant. The interview concludes with the questions, “Is there anything else you would like to share?” and a brief reflection on their experience of participating in the research.
    For older adults, the interview begins with questions about their background and life story to facilitate comfort and contextual understanding. It then gradually transitions into questions related to their well-being, and life experiences. The interview concludes with an invitation for overall reflections on their life and personal sense of well-being, as well as feedback on the interview experience.
- Ethics - The current study is approved by the Research Ethics Committee (CERec) of the Universitat Autònoma de Barcelona. The ethical standards of the study will be ensured with the assistance of Dr. Ruth Angiel from the Department of Applied Psychology at the Central University of Tamil Nadu, India, during the research period in India. Interviews will be conducted on a one-to-one basis in settings that are comfortable and familiar to participants, ensuring their sense of ease and safety.
  Prior to participation, individuals will be informed about the purpose and nature of the study and interview, their right to withdraw at any stage (including after providing consent), their freedom to respond only to questions they feel comfortable answering, and the measures taken to ensure confidentiality. Participation will be entirely voluntary, and interviews will proceed only after written informed consent has been obtained.
  Only essential demographic information will be collected at the beginning of the interview for contextual purposes, and this information will remain anonymous in all public outputs. To protect participants’ identities, all data will be pseudonymised by assigning a unique personal identification code to each participant. Two separate files will be maintained: one containing the research data without personal identifiers and another linking identification codes to participants’ names. The key linking file will be securely stored and accessible only to the doctoral researcher and supervising academic.
  Pseudonymisation is employed rather than full anonymisation due to the cross-cultural qualitative nature of the study, where limited traceability is necessary to ensure translation accuracy, contextual interpretation, and potential participant validation (e.g., follow-up clarification or member checking). Once data analysis and validation procedures are completed, the key linking file will be permanently deleted, resulting in full anonymisation of the dataset.
  All data will be securely stored in restricted-access institutional systems. Following completion of the study, the database will be closed, and all code tables will be securely deleted. The data will then be reviewed to confirm that the risk of re-identification is eliminated, and any additional measures required to ensure data security will be implemented.
- Pilot testing - A pilot phase was conducted to assess the clarity, flow, feasibility, and conceptual alignment of the semi-structured interview guides. Four pilot interviews were carried out—two with stakeholders and two with older adults.
  The pilot phase was instrumental in refining the interview guides. The initial pool of approximately twenty questions was reduced to ten core questions for each group to enhance conceptual focus, minimize redundancy, and ensure sufficient depth within each domain. Many of the original questions were overlapping, repetitive in concept, and directly asked about the themes. The revised approach framed questions so that they could naturally elicit insights aligned with the domains. Revisions also improved clarity, sequencing, and the coherence of thematic progression.
  The pilot interviews also helped evaluate whether the questions were comprehensible, contextually appropriate, and capable of eliciting rich and meaningful responses. Furthermore, the pilot confirmed the importance of maintaining flexibility in conducting semi-structured interviews. Allowing the interviewer to adapt the flow of questioning based on participants’ narratives facilitated a more natural progression of themes and supported deeper reflective engagement.
- Main interviews - A total of 10–13 main interviews will be conducted with each participant group in each country. Interviews with stakeholders will be conducted primarily in English in both India and Spain; however, if necessary, two to three interviews in India may be conducted in Malayalam, the regional language of the state of Kerala. Interviews with older adults in India will be conducted in Malayalam, while Interviews with older adults in Spain will be conducted in Spanish with the assistance of a fluent Spanish speaker. The assistant will be acting as an interpreter during the interview process. The interpreter will preferably have a background in psychology and will be briefed regarding the objectives of the study, ethical considerations, and the importance of maintaining a supportive, respectful, and participant-centered interview environment.
  The principal researcher will remain actively involved throughout all interviews, leading the interview process, engaging directly with participants, and making observational notes. Whenever possible, interviews will be conducted directly with Spanish older adults who are fluent in English, thereby minimizing the need for interpretation.
  To further enhance participant comfort and facilitate rapport, participants will be informed about the roles of both the researcher and interpreter prior to the interview. In addition, sufficient time will be allocated for introductions and informal conversation before the commencement of data collection.”
  All interviews will be audio-recorded for verbatim transcription using an in-built phone recorder, following informed consent from participants. The audio files will be subsequently transferred to a secure drive associated with the Universitat Autònoma de Barcelona (UAB). All data will be stored securely in the UAB OneDrive system, with access restricted to duly authorized personnel only. Confidentiality will be strictly maintained: personal identifiers will not be disclosed publicly, and data will be used exclusively for research purposes. Each participant will be assigned a unique personal identification number to ensure anonymity and protect the confidentiality of the data.

## 3 Discussion

The study of healthy aging has often been dominated by an objective biomedical approach, which overemphasizes disease-free conditions and can result in a discriminatory framework that excludes many older adults (Carver and Buchanan, 2016). Meanwhile, the literature review by Zamudio-Rodríguez et al. (2021) highlights discrepancies in aging research by pointing to inconsistencies between lay personal perspectives and biomedical and psychosocial models of healthy aging. The scoping review conducted by Cesari et al. (2024) on research gaps in healthy aging research further emphasizes the need for studies that are closer to implementation and that employ outcomes more meaningful to individuals, such as quality of life and well-being.

In this context, the present study gains significance as it seeks to address the gap in the literature, which often lacks comprehensive qualitative exploration within cultural contexts, particularly studies that integrate both older adults’ lived experiences and stakeholders’ perspectives (Ayoubi-Mahani et al., 2023). To our knowledge, this is the first study to explore healthy aging through a cross-cultural comparison between India and Spain. The present study aims to shift the conceptual understanding of healthy aging by exploring it as a process of living well throughout the lifespan, integrating the concepts of flourishing and quality of life. This perspective expands the understanding of aging beyond a predominantly biomedical condition to an optimistic, person-centered, and non-pharmacological approach to aging well.

By integrating the lived experiences of older adults with stakeholders’ perspectives based on their engagement with older populations, the study seeks to bridge the gap between lay personal understandings and existing models of healthy aging. In doing so, it aims to identify strategies and interventions that are meaningful and relevant to older adults. The study further provides insights into areas of convergence and divergence between older adults’ lived experiences and stakeholders’ perspectives. The cross-cultural design employed in the study facilitates the identification of both shared and culturally specific factors that contribute to flourishing, quality of life, and cognitive well-being in late adulthood. These insights can support the development of proactive and culturally sensitive programs, policies, and practices aligned with the World Health Organization’s healthy aging framework. Overall, the study contributes to reframing aging as a process of “growing old well,” emphasizing well-being and quality of life across the lifespan.

Qualitative research is particularly well suited for in-depth exploration beyond operationalized definitions of successful aging, as it captures the subjective meanings and lived experiences of older adults (Reichstadt et al., 2010). In addition, cross-cultural research in healthy aging has demonstrated that cultural contexts significantly shape priorities, values, and expectations related to aging. For instance, Sulandari et al. (2024) highlight how cultural settings influence perceptions of aging well, reinforcing the importance of cross-cultural perspectives in redefining healthy aging. The use of RTA further strengthens the methodological approach by enabling a rich, systematic, and detailed exploration of patterns within qualitative data (Braun and Clarke, 2006).

Despite the broad scope and potential contributions of this study, several limitations should be acknowledged. First, the findings cannot be generalized across all cultural contexts, as the participants were drawn from specific regions within India and Spain, which may limit the representativeness of the wider cultural populations in these countries. Second, as a qualitative study, the research prioritizes depth and richness of understanding over statistical generalizability, thereby limiting population-level inferences. Finally, although researcher reflexivity was employed to enhance the trustworthiness of the findings, future research could strengthen qualitative rigor by incorporating additional strategies and multiple rigor-enhancing techniques.

Although the present study will provide qualitative evidence that contributes to a more holistic model of healthy aging by integrating the human essence of aging beyond biomedical indicators. By foregrounding lived experiences of older adults alongside expert perspectives, the study elucidates areas of convergence and divergence between personal and professional understandings of aging well. The findings will offer insight into what older adults themselves value in later life, thereby supporting the development of more person-centered and culturally responsive healthy aging policies. The study also proposes non-pharmacological, optimistic, and psychosocial strategies for living well in later years, while highlighting opportunities for proactive measures across the lifespan. Additionally, the findings may support practitioners in moving beyond deficit-based models toward strength-based, flourishing-oriented approaches to care, and inform the enhancement of intergenerational and community-based programs.

## 4 Dissemination

The findings of this study will be disseminated to both academic and non-academic audiences, including the wider public, to translate research insights into practical implications. Academic dissemination will occur through peer-reviewed journal publications and presentations at national and international conferences.

To reach non-academic audiences, findings will be shared through the Marie Curie COFUND TOUCH social media platforms and the researcher’s professional LinkedIn profile. Additional dissemination will take place through the communication channels of the Foundation for Health and Aging. Research insights will also be communicated to the public through news articles and magazines.

All dissemination activities will be conducted in a manner that strictly maintains participants’ anonymity and confidentiality.
